# Hypotheses, rationale, design, and methods for prognostic evaluation of a randomized comparison between patients with coronary artery disease associated with ischemic cardiomyopathy who undergo medical or surgical treatment: MASS-VI (HF)

**DOI:** 10.1186/s13063-020-04270-w

**Published:** 2020-04-16

**Authors:** Paulo Cury Rezende, Whady Hueb, Edimar Alcides Bocchi, Michael Farkouh, Carlos Vicente Serrano Junior, Eduardo Gomes Lima, Expedito Eustáquio Ribeiro Silva, Luis Alberto Oliveira Dallan, Fabio Antonio Gaiotto, Cibele Larrosa Garzillo, Carlos Eduardo Rochitte, Cesar Higa Nomura, Thiago Luis Scudeler, Paulo Rogério Soares, Fabio Biscegli Jatene, José Antonio Franchini Ramires, Roberto Kalil Filho

**Affiliations:** 1 Instituto do Coraçao (InCor), Hospital das Clínicas HCFMUSP, Faculdade de Medicina, Universidade de São Paulo, Av. Dr. Eneas de Carvalho Aguiar 44, AB, Sala 114, Cerqueira César, São Paulo, SP 05403–000 Brazil; 2 Toronto General Hospital Research Institute (TGHRI), Toronto, Ontario Canada

**Keywords:** Ischemic cardiomyopathy, Ventricular dysfunction, Coronary artery disease, CABG, Randomized controlled trial

## Abstract

**Background:**

Ischemic cardiomyopathy and severe left ventricular dysfunction are well established to represent the main determinants of poor survival and premature death compared with preserved ventricular function. However, the role of myocardial revascularization as a therapeutic alternative is not known to improve the long-term prognosis in this group of patients. This study will investigate whether myocardial revascularization contributes to a better prognosis for patients compared with those treated with drugs alone and followed over the long term.

**Methods:**

The study will include 600 patients with coronary artery disease associated with ischemic cardiomyopathy. The surgical or drug therapy option will be randomized, and the events considered for analysis will be all-cause mortality, nonfatal infarction, unstable angina requiring additional revascularization, and stroke. The events will be analyzed according to the intent-to-treat principle. Patients with multivessel coronary disease and left ventricular ejection fraction measurements of less than 35% will be included. In addition, myocardial ischemia will be documented by myocardial scintigraphy. Markers of myocardial necrosis will be checked at admission and after the procedure.

**Discussion:**

The role of myocardial revascularization (CABG) in the treatment of patients with coronary artery disease and heart failure is not clearly established. The surgical option of revascularizing the myocardium is a procedure designed to reduce the load of myocardial hibernation in patients with heart failure caused by coronary artery disease. On the other hand, the assessment of myocardial viability is frequently used to identify patients with left ventricular ischemic dysfunction in which CABG may add survival benefit. However, the effectiveness of this option is uncertain. The great difficulty in establishing the efficacy of surgical intervention is based on the understanding of viability without ischemia. Thus, this study will include only patients with viable and truly ischemic myocardium to correct this anomaly.

**Trial registration:**

Evaluation of a randomized comparison between patients with coronary artery disease associated with ischemic cardiomyopathy submitted to medical or surgical treatment: MASS-VI (HF), ISRCTN77449548, Oct 10th, 2019 (retrospectively registered).

## Background

The Coronary Artery Surgery Study (CASS) was the pioneer in randomly identifying those patients with stable coronary artery disease (CAD) and preserved ventricular function who have a favorable prognosis. In addition, it reported that clinical and surgical treatments had a similar mortality occurrence in long-term follow-up [1]. Subsequent studies, such as the Veterans Administration Cooperative Study Group (VA) [2] and the European Coronary Surgery Study Group (European) [3], have found similarities in results relative to clinical or surgical treatments. However, unlike the CASS study, these studies included non-similar patients with unstable angina (European) and compromised ventricular function including ventricular aneurysms in the study sample. In addition, they observed increased incidences of death and nonfatal infarction in certain subgroups of patients [4]. These differences were attributed to the selection of patients with different degrees of clinical presentation and arterial and ventricular impairment. In fact, CASS, through a sub-analysis, identified that patients with ventricular dysfunction in clinical treatment evolved with increased mortality compared with those with preserved ventricular function undergoing myocardial revascularization [5].

Similarly, the Bypass Angioplasty Revascularization Investigation (BARI), which studied patients with preserved ventricular function, found a worse prognosis in patients with diabetes mellitus who underwent percutaneous treatment [6]. On the other hand, subsequent studies in diabetic patients and preserved ventricular function found no differences in mortality compared with the different therapeutic forms of CAD [7, 8]. In this scenario, the Stich Trial [9] was designed to compare only patients with multi-arterial CAD and ventricular dysfunction in different therapeutic forms. In our study, we did not find significant survival-related differences between clinical treatments and surgical revascularization, thus adding doubts to the understanding of the prognosis of the interventional treatment of ventricular dysfunction. Notably, the Stich Trial included patients with ventricular dysfunction but without the presence or absence of myocardial ischemia being identified.

This purpose of this study was to evaluate, in a randomized model, the results of surgical or medical treatments in patients with coronary artery disease and left ventricular dysfunction secondary to ischemic cardiomyopathy.

This is a prospective randomized clinical trial, with a 1:1 allocation ratio, designed to test the superiority of coronary artery bypass surgery over medical therapy alone in patients with coronary artery disease and left ventricular dysfunction secondary to ischemic cardiomyopathy.

## Methods/Design

**Fig. 1:**
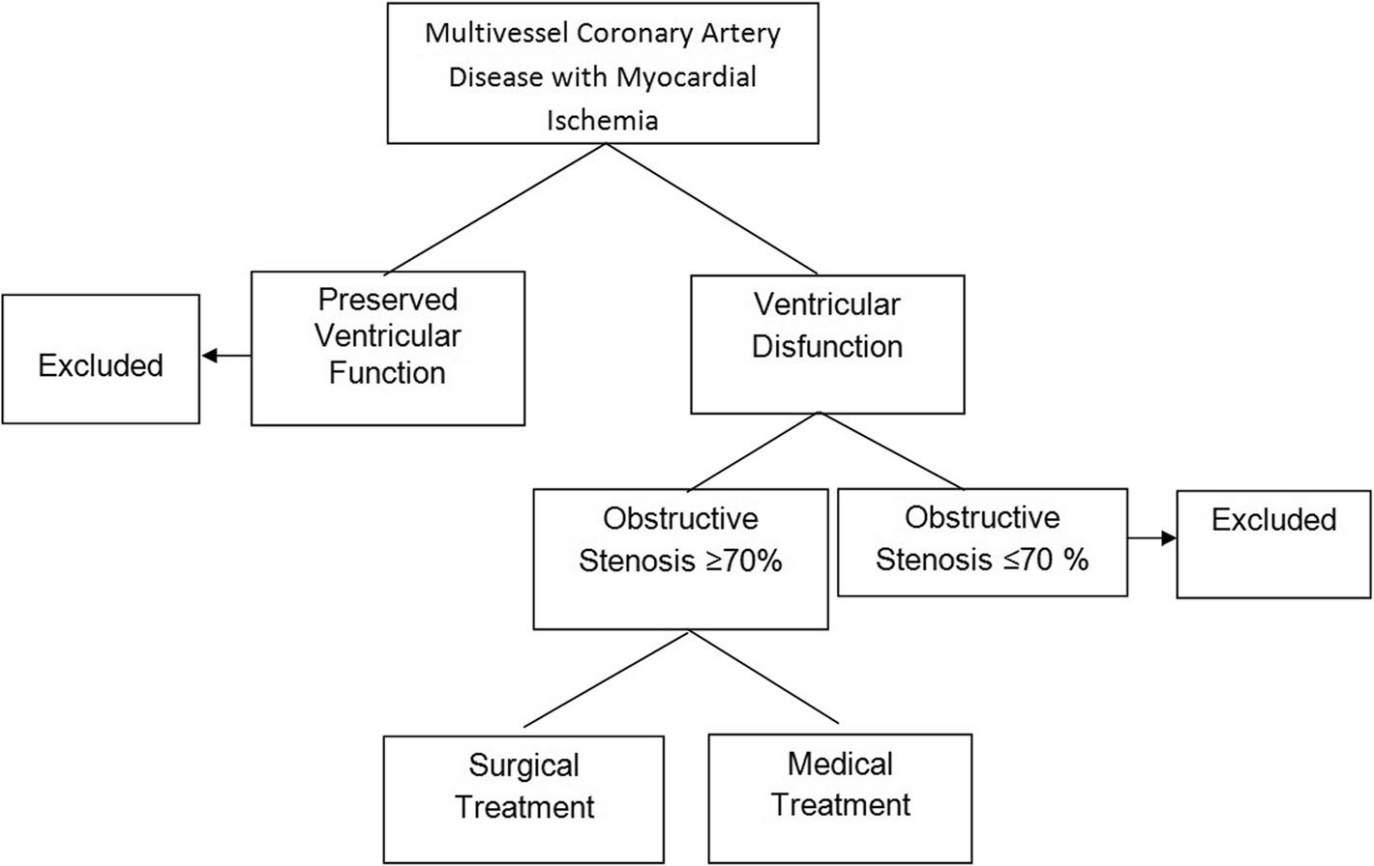
Patient selection flowchart

A total of 600 patients with angina pectoris will be screened for conventional coronary artery disease with obstructive lesions ≥ 70% of the arterial lumen and left ventricular dysfunction; 300 patients will be randomized to myocardial revascularization surgery, and another 300 patients will receive drug treatment. Patients who have been victims of acute myocardial infarction (AMI) in the previous 6 months, are on dialysis, or have disabling degenerative disease will not be included. In addition, patients who refuse to sign a consent form or who do not accept a possible surgical randomization will not be included. The stages of randomization are described in Fig. 1 (Patient selection flowchart). This is a study performed at an Academic Hospital. All data for inclusion, follow-up, and occurrence of events will be collected in a single center in Brazil.

### Surgical treatment

Patients randomized to surgery should have ventricular dysfunction with ejection fraction ≤ 35% and have lesions in multiple arteries. Surgery should be performed with the aid of extracorporeal circulation in all patients. In this procedure, the myocardial protection should be made with a standardized cardioplegic solution with a temperature close to 35 °C. Native vessels may receive venous grafts or arterial anastomoses at the discretion of the surgeon. Chronically occluded arteries may receive associated arterial or venous grafts. Obstructed artery endarterectomy may be the surgeon’s option. In addition to surgical intervention, patients will receive full medication for CAD as well as rigorous control of risk factors.

### Medical treatment

Both trial arms will be placed into optimal medical therapy that will include maximum tolerated beta-blockers, angiotensin-converting enzyme inhibitors or angiotensin receptor blockers, spironolactone, and diuretics if necessary. Antiplatelets agents, statins, and lifestyle changes will also be prescribed in each study visit.

Patients randomized to drug therapy will undergo laboratory tests on admission that include markers of myocardial necrosis, renal function tests, and liver tests for the detection of lipid or glycemic disorders. All patients will receive specific medications to control anginal symptoms as well as associated illnesses. Lifestyle changes and strict control of risk factors will be emphasized. Multiprofessional teams will be available to control this condition.

### Laboratory tests

Patients undergoing surgery will have routine biochemical exams before and after surgery. In addition, C-reactive ultra-sensitive protein (CRP) will be checked immediately before and after surgery. In addition, biomarkers of myocardial necrosis will also be checked before and after the surgical procedure. Electrocardiography will be performed before and after the operation, and echocardiography will be performed before and after the operation for analysis of ventricular function.

### Myocardial scintigraphy

At study admission all patients will undergo scintigraphic examination for the detection of ischemic areas. The quality of the images and also the ischemic area under risk will be considered for analysis. For inclusion in the study, ≥ 10% of the area at risk will be considered.

### Echocardiography

Patients in the surgical group will undergo echocardiography immediately before the procedure, at hospital discharge, and 1 year after the procedure. Simpson’s biplanar method will be employed. In patients in the clinical group, echocardiography will be performed at admission, after 1 year, and at the end of follow-up.

### Primary endpoint of the study

Combined primary endpoints will be considered as those that occurred during the study: death from any cause, nonfatal myocardial infarction, stroke and unstable angina requiring additional intervention.

The definitions of each event are available in Table 1.

**Table 1:** Definitions of combined endpoints

**Mortality from any cause**	Mortality from any cause is included as a composite primary event. Cardiovascular death includes that from myocardial infarction, sudden death, refractory heart failure, fatal hemorrhagic cerebral infarction or fatal cerebral hemorrhage related to the procedure.
**Vascular brain accident**	Patients with focal neurological deficit of central origin lasting more than 72 h or a focal neurological deficit of central origin that lasted more than 24 h, with evidence of images of cerebral infarction or intracerebral hemorrhage, or non-focal encephalopathy that lasted more than 24 h, with evidence of images of cerebral infarction or bleeding sufficient to warrant clinical status. Retinal arterial ischemia or bleeding is also included. To meet the definitions of stroke, the deficit must be new or sudden—consensually—not attributable to any alternative.
**Acute myocardial infarction**	Elevation of specific cardiac enzymes within 14 days of the revascularization procedure and the presence of new Q waves in at least two or more contiguous ECG leads, and elevation of 10x the 99th percentile of CK-MB and ultrasensitive troponin.
**Additional revascularization**	Initial revascularization is considered complete when the patient is transferred from the operating room to the ward. Refractory angina requiring additional revascularization is considered an event.

### Secondary endpoint

The secondary endpoint, during the study follow-up, will be the graduation of anginal symptoms and also of heart failure. Cardiac decompensation hospitalization will be considered a secondary event

### Ambulatory follow-up

All patients will have periodic outpatient follow-up as described in Table 2. This follow-up will be independent of the treatment group to which the patient was allocated, and participants will be followed in close outpatient visits. All patients and families were given a telephone number of the study coordinating center in case of doubts or any clinical minor or major event. This close follow-up will permit good retention rates and a complete follow-up. Neurological evaluation will be available to patients after surgery and, if necessary, at follow-up. Cardiological evaluation will be done throughout the follow-up. No biological specimens will be collected in this study.

**Table 1:** Schedule of the study, with all detailed assessments for each study period

	Study period								
	Enrollment	Allocation	Post-allocation					Close- out	
Timepoint**	***Interview***	**Rand**	**1 M**	**6 M**	**12 M**	**24 M**	**36 M**	**48 M**	**60 M**
**Enrollment**	X								
**Eligibility screen**	X								
**Informed consent**		X							
**Allocation**		X							
**Angiograms**		X							
**History/events**	X	X	X	X	X	X	X	X	X
**Anginal assessment**	X	X	X	X	X	X	X	X	X
**Dyspnea assessment**	X	X	X	X	X	X	X	X	X
**Medicines**	X	X	X	X	X	X	X	X	X
**ECG**	X		X		X	X	X	X	X
**Echocardiography**	X		X						X
**Laboratory**	X				X	X	X	X	X
**Interventions:**									
***Surgery***			X						
***Angiograms***									X
**Assessments:**									
***Follow-up***			X	X	X	X	X	X	X
***CT angiography***									X
***Neuropsychological Tests***			X						X
***ECG stress tests***					X				X
***Quality of life***	X			X	X	X	X	X	X
***Costs and effectiveness***			X						X
***Working status***			X						X

*M* month, *ECG* electrocardiogram. Laboratory tests will include creatinine, LDL-cholesterol, glycated hemoglobin, CK-MB, troponin, and BNP levels

### Ancillary studies

The quality of life with application of the SF-36 questionnaire and the cost-effectiveness evaluation between the two types of treatment will be applied with outpatient follow-up for 5 years.

### Quality of life

Quality of life of treatment related to the health of patients will be evaluated in the entire population of the patients of MASS-VI (HF). Quality of life related to symptoms as well as the functional status of patients will be assessed through a combination of generic and disease-specific measures selected to cover a wide range of potential health domains that may be affected by coronary artery disease, their treatments, and complications. The SF-36 Questionnaire will be used to evaluate patient condition [10].

### Cost-effectiveness—working condition

The use of this resource using a global health classification that reflects a patient’s preference for his or her current health status in relation to perfect health are particularly important outcome measures for the cost-effectiveness analysis [11]. Use of resources and health care with hospital cost data will be collected prospectively from admission to discharge and remain for a 5-year follow-up for all patients, including all costs associated with the interventional procedure. For each revascularization procedure, the detailed use of resources will be collected through a standard case report form. Use of medical follow-up resources (including hospitalization, outpatient services, and cardiovascular medications) will be evaluated through detailed questionnaires that will be completed during each scheduled patient contact.

### Statistical analysis

Patients eligible for the MASS-VI (HF) study will be invited to attend the Heart Institute– FMUSP outpatient clinic to receive additional information. Candidates for surgical treatment when admitted will be visited by one of the study doctors. After giving consent, patients will be randomized. To ensure a reasonable balance of the sample, the assignment is made according to a computer-generated list of randomly permuted blocks that are unknown to the investigators. The generation of the allocation sequence is in the statistics department without the possibility of access to researchers.

After randomization, patients will be scheduled for the intended treatment. The design is open label with only outcome assessors being blinded, so unblinding will not occur. The objective of the main analysis is to compare the results of the primary events of this study. Kaplan-Meier curves will be used for graphical comparison of the data. All values will be expressed as mean ± standard deviation or percentage. The continuous variables will be compared by the Wilcoxon test, whereas the variables will be analyzed with the Fisher exact test. The occurrence of event results will be compared using the Cox proportional hazards model, reflecting a risk rate. The analysis of the primary objectives will be based on the intention-to-treat principle. Interim analyses will be conducted annually to assess safety. For both treatment strategies, the cost of diagnostic procedures, treatment procedures, complications, and short- and long-term effect differences will be estimated. Marginal costs in monetary terms will be calculated by multiplying unit costs and marginal medical consumption as recorded for each patient.

### Sample size calculation

Based on the event rates previously reported in the STICH trial [9], and assuming that the primary endpoint defined for this study will be of 9% in the medical therapy arm, the number of patients that needs to be included is 585, with an alfa error of 5% and a beta of 20%.

## Discussion

Myocardial ischemia accompanied by ventricular dysfunction is assumed to play a relevant role in the occurrence of anginal symptoms and symptoms of heart failure and correcting this condition is assumed to promote the relief of symptoms. Assuming that aorto-coronary leads or arterial grafts play a relevant role in the abolition of these clinical symptoms is intuitive. For this to occur, anatomical conditions of the native arteries must be adequate to receive the grafts, thus allowing good blood flow distal to the lesion. These conditions are found in patients with coronary lesions of proximal location that allow a suitable blood pressure gradient for a good distal flow. However, ventricular dysfunction caused by hibernating myocardium does not always have the benefits of surgical revascularization because of the presence of residual fibrosis to the old myocardial infarction. Given this, frequently, patients under these conditions have surgery denied because of possible inexpressive or even adverse results. In this scenario, the option for percutaneous treatment is impaired for the same reason because the technique of multiple percutaneous artery interventions has unfavorable results and is not exempt from immediate risks. Thus, the option for drug treatment is posed by the absence of a surgical alternative. Moreover, few comparative studies are aimed at answering this question. Finally, even though surgical intervention is an important option, a final comparison with the results of drug treatment may lead to a safer decision.

### Final considerations

The project “MASS-VI (HF)” was developed to include patients with angina pectoris and dyspnea with severe impairment in ventricular function. Because of this dysfunction, the treatment with medication was established as the only therapeutic option because myocardial revascularization surgery would be a complex option and with uncertain results. The results of the study may facilitate the choice of the most appropriate strategy for each patient and promote the appropriate use of available resources.

## Trial status

The review of the literature has started, and the search queries for the databases have been established. Currently, the investigators are performing the screening. The current version is number 1, dated March 26, 2019.

At the time of first submission, the trial had enrolled three patients. Recruitment started in July 2019 and is expected to continue until the middle of 2024.

## Data Availability

The datasets analyzed during the current study are available from the corresponding author on reasonable request.
